# Phylogenomic Analyses Show Repeated Evolution of Hypertrophied Lips Among Lake Malawi Cichlid Fishes

**DOI:** 10.1093/gbe/evac051

**Published:** 2022-04-13

**Authors:** Paul Masonick, Axel Meyer, Christopher Darrin Hulsey

**Affiliations:** Department of Biology, University of Konstanz, Universitätsstraße 10, 78464 Konstanz, Germany

**Keywords:** whole-genome resequencing, mbuna, parallelism, coding vs. noncoding

## Abstract

Cichlid fishes have repeatedly evolved an astounding diversity of trophic morphologies. For example, hypertrophied lips have evolved multiple times in both African and Neotropical cichlids and could have even evolved convergently within single species assemblages such as African Lake Malawi cichlids. However, the extremely high diversification rate in Lake Malawi cichlids and extensive potential for hybridization has cast doubt on whether even genome-level phylogenetic reconstructions could delineate if these types of adaptations have evolved once or multiple times. To examine the evolution of this iconic trait using protein-coding and noncoding single nucleotide polymorphisms (SNPs), we analyzed the genomes of 86 Lake Malawi cichlid species, including 33 de novo resequenced genomes. Surprisingly, genome-wide protein-coding SNPs exhibited enough phylogenetic informativeness to reconstruct interspecific and intraspecific relationships of hypertrophied lip cichlids, although noncoding SNPs provided better support. However, thinning of noncoding SNPs indicated most discrepancies come from the relatively smaller number of protein-coding sites and not from fundamental differences in their phylogenetic informativeness. Both coding and noncoding reconstructions showed that several “sand-dwelling” hypertrophied lip species, sampled intraspecifically, form a clade interspersed with a few other nonhypertrophied lip lineages. We also recovered *Abactochromis labrosus* within the rock-dwelling “mbuna” lineage, starkly contrasting with the affinities of other hypertrophied lip taxa found in the largely sand-dwelling “nonmbuna” component of this radiation. Comparative analyses coupled with tests for introgression indicate there is no widespread introgression between the hypertrophied lip lineages and taken together suggest this trophic phenotype has likely evolved at least twice independently within-lake Malawi.

**Significance**

Convergent evolution is widespread in nature. Although this phenomenon is known to occur across cichlid fishes found in different parts of the world, in this study we used genome-wide SNPs to resolve whether a specialized trophic morphology, hypertrophied lips, evolved convergently among cichlids endemic to Lake Malawi. Our analyses provided well-supported inferences of relationships even within closely related species and showed that hypertrophied lips likely evolved at least twice independently within the two major radiations of Lake Malawi cichlids.

## Introduction

Evolution does repeat itself and convergently evolved adaptations speak to the nonrandomness of phenotypic evolution. As species diversify, ecological selective pressures could commonly result in replicate evolution within divergent lineages and drive them to converge on similar adaptive phenotypes. Convergent evolution is an especially common feature of adaptive radiations. For instance, Darwin’s finches (Grant and Grant 2008), Caribbean *Anolis* lizards (Losos et al. 1998), and three-spined stickleback fishes (Rundle et al. 2000; Marques et al. 2017) all have repeatedly evolved the same structural modifications. Most of this replicate evolution, whether it occurred over long timeframes independently or rapidly through convergence, has been documented in different geographic localities. The exceptionally diverse radiations of cichlid fishes offer classic examples of these allopatrically derived convergent phenotypes (Kocher et al. 1993; Meyer 1993; Stiassny and Meyer 1999; Salzburger et al. 2005; Salzburger 2009; Elmer and Meyer 2011; Muschick et al. 2012; Kratochwil et al. 2018; Ronco et al. 2021). However, parallelism can also occur within the same geographic region and even within the same closely related lineage (Elmer et al. 2010; Torres-Dowdall and Meyer 2021). Yet, when similar phenotypes arise in the same geographic locations over short timeframes, especially in highly diverse radiations, it is difficult for even genome-wide data to evaluate whether traits have arisen only once, evolved independently, originated in parallel, been retained as ancient polymorphisms, or are shared among taxa due to hybridization (Hulsey et al. 2019; Kautt et al. 2020). To evaluate between these evolutionary alternatives, that is, whether a particular adaptive phenotype arose once or multiple times in a classic example of adaptive radiation, we evaluated the ability of both protein-coding and noncoding data obtained from whole-genome resequencing to delineate the evolution of hypertrophied lips in Lake Malawi cichlids.

Cichlids have acquired a huge diversity of trophic morphologies that are specialized for different feeding niches, and hypertrophied lips are one of the most easily recognized phenotypes that have evolved multiple times independently (Burress et al. 2013; Colombo et al. 2013; Manousaki et al. 2013; Henning and Meyer 2014; Baumgarten et al. 2015; Henning et al. 2017). This distinct trophic innovation found in both Neotropical and African cichlid lineages is typically associated with fish that forage in rocky substrates where the lips may act as a seal to help suck prey from in between narrow cracks and crevices (Ribbink et al. 1983; Baumgarten et al. 2015), absorb stress from repeated contact with hard and rough surfaces (Fryer and Iles 1972; Greenwood 1974), and/or enhance prey detection by providing an enlarged area for taste receptors (Oliver and Arnegard 2010). This morphology also exhibits strong feeding tradeoffs with hypertrophied lip fish being more efficient at extracting prey from crevices but less apt at capturing evasive prey in open water (Elmer et al. 2010; Colombo et al. 2013; Machado-Schiaffino et al. 2017). Lip size may not only be important in terms of natural selection, but also play a role in sexual selection because assortative mating based on lip size has been found in polymorphic populations (Machado-Schiaffino et al. 2014, 2017). Considerable plasticity in the trait has also been observed in the laboratory possibly hinting that this trait can be acquired and lost over short timeframes (Machado-Schiaffino et al. 2014, 2017). The relative ease of diagnosing this qualitative phenotype that has testable ecological consequences makes it a model trait for studying adaptive evolution and convergence.

The hypertrophied-lip phenotype is also one of the iconic examples of a trait that has arisen independently in all three major cichlid adaptive radiations that are endemic to East Africa’s largest great lakes. This specialized morphology is found in *Haplochromis chilotes* from Lake Victoria, *Lobochilotes labiatus* from Lake Tanganyika, as well as eight species (including one undescribed species) native to Lake Malawi (Fryer and Iles 1972; Oliver and Arnegard 2010). Given the recurrent evolution of hypertrophied lips across these major East African cichlid lineages, it is plausible that the phenotype has also evolved independently multiple times in Lake Malawi (Hulsey et al. 2018). With roughly 850 species of haplochromine cichlids inhabiting this large African lake (fig. 2), the opportunity for adaptive traits to arise repeatedly in Malawi is considerable (Danley and Kocher 2001; Genner and Turner 2005; Konings 2007). The vast majority of Lake Malawi endemic cichlids belong to the tribe Haplochromini, but in this lake, have traditionally been placed in two main lineages: the primarily rock-dwelling mbuna (Genner and Turner 2005) and the largely sand-dwelling nonmbuna (Danley and Kocher 2001). These major ecomorphological groups have also consistently been recovered as distinct clades in molecular phylogenetic analyses (Meyer et al. 1990, 1994, 1996; Meyer 1993; Hulsey et al. 2017, 2018; Malinsky et al. 2018). Additionally, all but one species of hypertrophied-lip taxa are classified as nonmbuna haplochromines and have been assorted into different genera largely on the basis of body pigmentation patterns (Fryer and Iles 1972; Arnegard and Snoeks 2001; Snoeks 2004; Konings 2007). The one putative mbuna hypertrophied-lip species, *Abactochromis labrosus*, is an evolutionarily enigmatic cichlid (Trewavas 1935; Eccles and Trewavas 1989; Oliver and Arnegard 2010). Only a single phylogenetic study has included *A. labrosus* along with a limited number of taxa (15 Lake Malawi species in total) and this was based solely on mitochondrial control region sequences that inferred this species split at the base of the mbuna radiation (Meyer et al. 1996). Hypertrophied lips provide a readily diagnosable and potentially phylogenetically labile phenotype that could provide a more general model to test alternative hypotheses about what we can infer regarding how novel traits tend to evolve in Lake Malawi.

Previous work with mitochondrial DNA, nuclear AFLP loci, and ultraconserved elements (UCEs) have all repeatedly highlighted the issues in obtaining a clear phylogenetic consensus for the Malawi radiation (Kocher et al. 1995; Salzburger and Meyer 2004; Hulsey et al. 2010, 2013, 2018; Joyce et al. 2011). In seeking to address whether the hypertrophied-lip phenotype originated multiple times among Lake Malawi cichlids, Hulsey et al. (2018) evaluated the relationships of hypertrophied-lip taxa from several genera by analyzing single nucleotide polymorphisms (SNPs) from UCEs. Their results suggested that *Cheilochromis euchilus*, *Eclectochromis ornatus*, *Placidochromis milomo*, and *Placidochromis* “Mbenji fatlip”, all taxa that forage along rocky shores (Froese and Pauly 2021), along with several normal-sized lip species form a clade and that this remarkable phenotype and trophic guild originated just once among nonmbuna haplochromines (Hulsey et al. 2018). Conversely, the disparate placement of hypertrophied-lip species in reconstructions based on whole-genome sequences suggests that the enlarged lip condition either evolved more than once or possibly reverted to the ancestral condition in several closely related lineages (Malinsky et al. 2018). However, a lack of intraspecific sampling with respect to these taxa in that study limited what can be inferred regarding their species-level relationships. Further, these previous studies contrasted with findings from earlier analyses based on mitochondrial data that found these hypertrophied-lip taxa to be highly polyphyletic with *P. milomo* inferred to be nested even within the mbuna (Hulsey et al. 2010; Joyce et al. 2011).

Phylogenomic studies have traditionally relied more heavily on coding sequences due to the relative ease of PCR-amplification and the ease of identifying orthologous sequences in conserved amino acids that facilitates straightforward alignment of homologous sequences (Townsend et al. 2008; Thomson et al. 2010). However, when compared with other faster-evolving noncoding regions, the more conserved nature of coding sequences may limit the ability to resolve the evolutionary history of recent and rapid adaptive radiations (Meyer 1994; El Taher et al. 2021). Further, coding sequences could more often be functionally constrained, potentially prone to convergent evolution, and carry signal incongruent with the true species tree (Steinke et al. 2006; Parker et al. 2013). In contrast, noncoding regions are generally less susceptible to convergence, exhibit greater variability because of their faster substitution rates, and are informative at shallow evolutionary timescales (Meyer et al. 1990; Chojnowski et al. 2008; Yu et al. 2011; Foley et al. 2015). Given these factors, noncoding data may provide more robust phylogenetic signal than coding sequence data in a rapid radiation like the Lake Malawi cichlids and be especially useful for parsing the number of times traits like hypertrophied lips have evolved (Chen et al. 2017). However, in the era of whole-genome resequencing, a distinction between using coding and noncoding DNA for phylogenetics could seem extraneous because both can be readily obtained from the same completely sequenced genomes. Nonetheless, there is an ever-increasing ability to combine genome-wide data with subsets of the genome such as RAD-tag markers or transcriptome sequences (Sharma et al. 2014; Rahman et al. 2021). Transcriptome sequences for instance primarily produce information about protein-coding sequences and a heavy reliance on this component of the genome could be problematic (Lemmon and Lemmon 2013; Yang and Smith 2014; Cheon et al. 2020; Smith and Hahn 2021). Distinguishing the relative ability of coding and noncoding sequences to resolve phylogenetic relationships at various stages of diversification among Lake Malawi cichlids could inform not only future sequencing strategies but influence inferences concerning phenotypic convergence.

Robust species tree reconstructions play a crucial role in testing for convergence and one of its most powerful uses is to reveal whether phenotypically similar traits have originated once or multiple times (Omland 1999; Revell 2012). Nevertheless, our knowledge of Malawi cichlid relationships and ability to draw meaningful conclusions regarding the evolution of the group has long been impeded by the limited ability of molecular markers to provide phylogenetic resolution (Salzburger and Meyer 2004, Hashem et al. 2020). A number of factors that have complicated the phylogenetic reconstruction of Malawi cichlids are also shared with the even faster-evolving Lake Victoria cichlid radiation. These factors include the impressive phenotypic diversity of these fishes, the recent ages of the radiations (∼2 million years old and perhaps as young as ∼0.4 Ma) (Hulsey et al. 2010; Friedman et al. 2013; Genner et al. 2015; Meyer et al. 2017), exceptionally low interspecific genetic divergence (Meyer et al. 1990; Malinsky et al. 2018) rampant incomplete lineage sorting (Moran and Kornfield 1993), and the high potential for hybridization within the clades (Mims et al. 2010; Brawand et al. 2014). In both Lakes Victoria and Malawi, all of these factors could make resolving whether a trait like hypertrophied lips has originated multiple times intractable.

Additionally, even if phylogenetic inference typically supports the nonmonophyly of a trait such as hypertrophied lips, it is difficult to discount that a trait might have only evolved a single time. This is because traits might also have arisen once but be lost multiple times thereby appearing to have evolved repeatedly. The evolutionary loss of eyes in cave-adapted fishes (Coghill et al. 2014), flightlessness in island birds (Wright et al. 2016), and lack of terrestrial adult stages in neotenic salamanders (Riley et al. 2003; Bonett et al. 2021) all have likely occurred multiple times and should not lead to the erroneous inference that distinctive and complex traits such as eyes, wings, or adult forms evolved multiple times. Therefore, even the best phylogenetic reconstructions of trait evolution make it difficult to ascertain how many times a trait was gained and lost among exceptionally closely related taxa. Furthermore, despite the fact that traits might seem to have arisen repeatedly in more phylogenetically disparate groups, introgression can play a role in the phylogenetic distribution of these “convergent” phenotypes (Stern 2013).

**Fig. 1.**
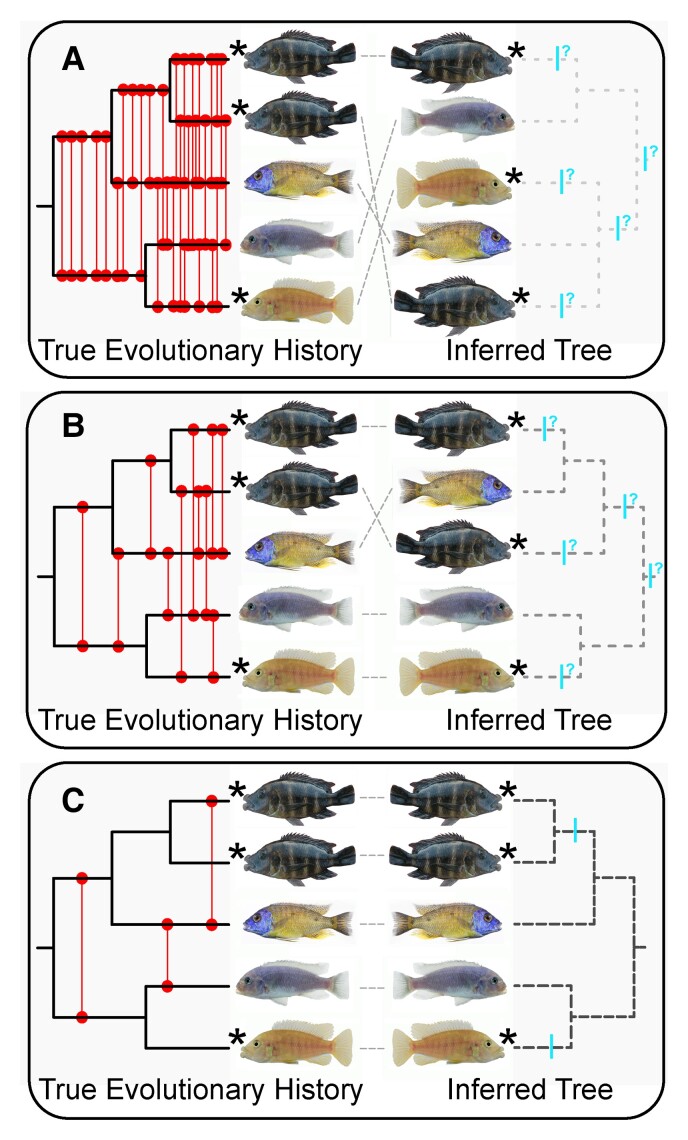
Phylogenetic reconstruction and our ability to infer convergence could be impacted heavily by genomic introgression. Illustrated are three hypothetical scenarios showing the extent that introgression could affect tree inference in the Lake Malawi cichlid radiation. In each panel, the true evolutionary history (the topology of which remains the same across *A*–*C*) is depicted on the left and the resulting phylogenetic reconstruction influenced by different degrees of hybridization is shown on the right. Taxa possessing hypertrophied lips are indicated with stars. Red lines denote prior hybridization events and blue bars represent possible origins of the hypertrophied-lip phenotype. Incongruence between the true history and the reconstructed tree is shown with gray dashed lines. In (*A*), phylogenetic resolution is completely obscured by factors such as widespread hybridization and incomplete lineage sorting resulting in the failure to recover conspecifics as monophyletic groups. Convergence of the hypertrophied-lip phenotype cannot be tested with bifurcating phylogeny-based comparative methods under this scenario. In (*B*), some phylogenetic structure is detectable, but clarity is insufficient to resolve many relationships among hypertrophied-lip species. Convergence is difficult to examine due to introgression and the inability to recover conspecifics as monophyletic. (*C*) Despite some gene flow, phylogenetic structure is clearly resolved, conspecifics are recovered as monophyletic, and there is a clear indication that hypertrophied lips are independently derived in disparate lineages.

Inferring whether convergent phenotypes have evolved could be impacted substantially by the degree of introgression that has occurred within the Lake Malawi radiation (fig. 1). Given that many lineages of Malawi cichlids do hybridize (Stauffer et al. 1996; Streelman et al. 2004; Mims et al. 2010), interspecific gene flow even across evolutionarily disparate lineages could play a large role in the phylogenetic distribution and putative independent evolution of a trait like hypertrophied lips. In addition to the difficulties incomplete lineage sorting pose for phylogenetic inference, if genetic admixture was rampant during the diversification of Lake Malawi cichlids, tree-like phylogenetic signal may be too severely obscured to resolve relationships at any level and leave us unable to assess convergence in any traits (fig. 1*A*). Even with a better-resolved phylogeny, if gene flow was particularly extensive among hypertrophied-lip taxa, we might fail to recover conspecifics as monophyletic groups and could have difficulty inferring the number of origins of a trait (fig. 1*B*). Alternatively, if phylogenetic structure is clearly retraceable and conspecifics are found to be monophyletic even in the presence of low levels of hybridization (fig. 1*C*), we could obtain a clear indication of whether the hypertrophied-lip trait was likely independently derived in multiple lineages.

Whole-genome resequencing now offers the opportunity to resolve relationships among closely related Malawi cichlids and should provide the power to reveal cases of within-lake convergent evolution (Brawand et al. 2014; Conte et al. 2019). In this study, we employed whole-genome resequencing to explore the evolution of the hypertrophied-lip phenotype among Lake Malawi cichlids, evaluated the ability of SNPs from coding and noncoding regions to reconstruct these and other relationships at different tree depths, and tested whether substantial gene flow could explain the phylogenetic distribution of the hypertrophied-lip phenotype. To assess the evolutionary history of hypertrophied-lip taxa, we sampled *A. labrosus* along with four hypertrophied-lip nonmbuna species (three of which were represented by multiple intraspecific samples), additional lineages of normal-lipped sand-dwelling nonmbuna, and a range of rock-dwelling mbuna species. This broad taxonomic sampling enabled us to test (1) whether hypertrophied-lip nonmbuna species evolved repeatedly, (2) are closely related to each other, and (3) finally narrow down the enigmatic phylogenetic position of *Abactochromis* as possibly the only hypertrophied-lip mbuna. As it can be difficult to know whether traits evolved independently or via allele sharing and adaptive introgression, we also tested for interspecific gene flow across the radiation with a focus on hypertrophied-lip species within Malawi cichlids to see if large-scale introgression could readily explain any inferences of the phenotype’s repeated origins.

## Results and Discussion

### Phylogenomics of Hypertrophied Lips in Lake Malawi

**Fig. 2.**
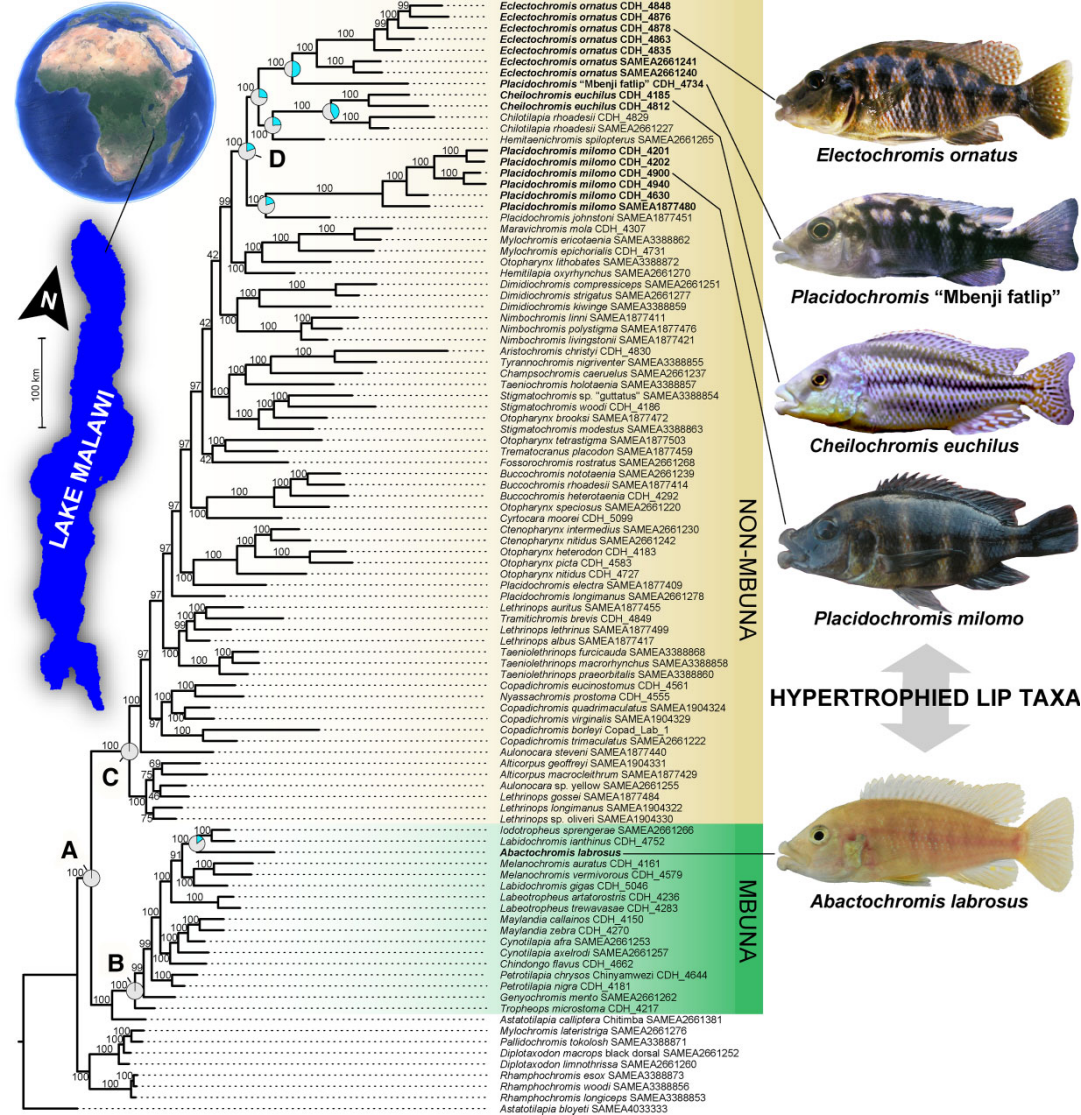
Species tree reconstruction of cichlids from African Lake Malawi based on maximum likelihood analysis in IQ-TREE of 1,107,249 noncoding SNPs. Ultrafast bootstrap support values are displayed for each branch. Taxa possessing hypertrophied lips, denoted in bold, are recovered in largely two positions in the phylogeny. Notably, intraspecific sampling of these species recovered monophyletic groupings. Pie-diagrams represent the probability that the ancestral condition for select nodes was hypertrophied lips (indicated by the upper right segments in blue). Node A: MRCA of all Lake Malawi hypertrophied species, node B: MRCA of the rock-dwelling mbuna radiation, node C: MRCA of the sand-dwelling nonmbuna, node D: MCRA of the nonmbuna hypertrophied-lip fauna. The five hypertrophied Lake Malawi species sampled are pictured to the right of the phylogeny.

**Fig. 3.**
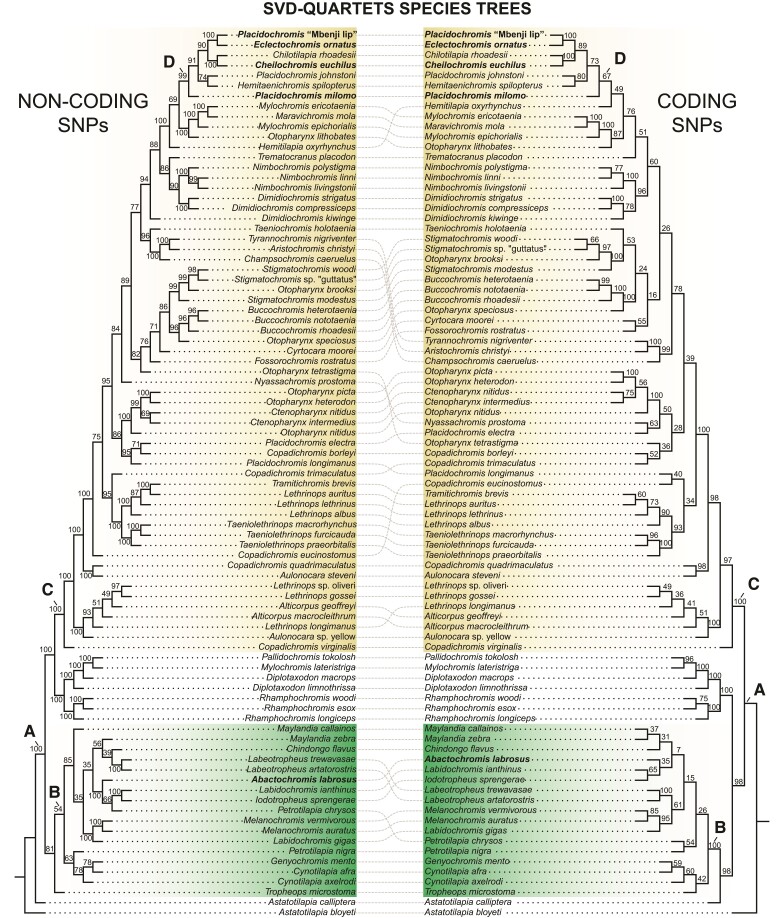
Relationships of Lake Malawi species based on analysis with SVDquartets. The reconstructions based on noncoding vs. coding SNPs are compared. Dotted lines are used to connect species that exhibit slightly divergent phylogenetic relationships based on the two data types. Bootstrap values are indicated for each branch. Node A: MRCA of all Lake Malawi hypertrophied species, node B: MRCA of the rock-dwelling mbuna radiation, node C: MRCA of the sand-dwelling haplochromine nonmbuna, node D: MCRA of the nonmbuna hypertrophied-lip fauna.

Our phylogenetic analyses are based on 1,352,537 SNPs derived from whole-genome resequencing of 86 Lake Malawi cichlid species that included 33 newly generated genome sequences (supplementary table S1, Supplementary Material online). We recovered robust phylogenetic hypotheses of Malawi cichlid relationships based on concatenation (IQ-TREE, Nguyen et al. 2015) and multispecies coalescent-based (SVDquartets—Chifman and Kubatko 2014) approaches that inferred largely similar species trees across both the noncoding and coding SNP datasets (figs. 2 and 3, supplementary fig. S1, Supplementary Material online). All analyses found distinct, well-supported clades containing the rock-dwelling mbuna and primarily sand-dwelling nonmbuna (nodes B and C, respectively figs. 2 and 3, supplementary fig. S1, Supplementary Material online: 100 UFBS/100 BS). Further, the hypertrophied-lip taxa were unambiguously resolved as polyphyletic (node A of figs. 2 and 3 represents the inferred MRCA of all hypertrophied-lip species). *Abactochromis labrosus* is clearly nested within the mbuna and shares a close affinity to the other rock-dwelling lineages *Labidochromis*, *Iodotropheus sprengerae*, and the two *Melanochromis* species examined. This result corroborates relatively recent taxonomic work that proposed that *A. labrosus* is distinct from species of *Melanochromis* but is still likely a member of the mbuna (Oliver and Arnegard 2010). However, the two *Labidochromis* species sampled are not monophyletic with *L. ianthinus* and *I. sprengerae* grouping together and *L. gigas* clustering with *Melanochromis*. The hypertrophied-lip taxa *C. euchilus*, *E. ornatus*, *P. milomo*, and *Placidochromis* “Mbenji fatlip” form a clade within the nonmbuna that also contains a few nonhypertrophied-lip species (*Chilotilapia rhoadesii*, *Hemitaenichromis spilopterus*, and *Placidochromis johnstoni*) (node D, figs. 1 and 2: 100 UFBS/99 BS [noncoding]/67 BS [coding]). *C. euchilus* and the normal-lipped *C. rhoadesii* are recovered as sister taxa in both the ML and SVDquartet reconstructions. Although *P. milomo* and *P. johnstoni* compose another group according to our IQ-TREE reconstruction, SVDquartets found *P. milomo* as the sister to the other hypertrophied-lip taxa + *C. rhoadesii*, *H. spilopterus*, and *P. johnstoni*. Our phylogenetic results support the notion that there has likely been repeated evolution of the hypertrophied-lip phenotype in Lake Malawi cichlids.

As we did not recover a group exclusively composed of hypertrophied-lip nonmbuna, the question remains as to whether enlarged lips arose once or multiple times among these taxa. We investigated this further with maximum likelihood-based ancestral state reconstruction. From this analysis, the ∼50% probability that the ancestor of any specific hypertrophied-lip species and its close relatives possessed enlarged lips suggests that there could have been multiple transitions to the hypertrophied-lip phenotype within this relatively small subclade of the nonmbuna (see reconstructions for lineages nested under node D, fig. 2, supplementary fig. S2, Supplementary Material online). An alternative explanation for this pattern would be that the ancestor of these lineages evolved hypertrophied lips and that there were multiple reversals back to the much more common normal-lip morphology (e.g., with possible reversals occurring in *P. johnstoni* and/or *C. rhoadesii*). In contrast to this relative ambiguity, the probability that the common ancestor of the nonmbuna hypertrophied-lip species and *Abactochromis* had hypertrophied lips is exceedingly low (0.1%—node A, fig. 2), and it is highly unlikely that the most recent common ancestor of the mbuna or nonmbuna exhibited this phenotype (0.5%—node B, 0.1%—node C, fig. 2). Based on our phylogenetic inferences coupled with ancestral state reconstruction, we can confidently conclude that there clearly are at least two independent origins of hypertrophied lips in the Lake Malawi cichlids (one in the mbuna and one in the nonmbuna).

However, as missing taxa may bias ancestral state reconstruction (Omland 1999; Salisbury and Kim 2001), any ancestral state reconstruction in this diverse radiation should be interpreted with caution. *Otopharynx pachycheilus*, *Lichnochromis acuticeps*, and *Trematocranus pachychilus* are three other Malawi haplochromine cichlids with enlarged lips that were not sampled in this study. However, according to previous analysis, *L. acuticeps* is closely related to the hypertrophied-lip nonmbuna (Hulsey et al. 2018). *Otopharynx* represents another genus of nonmbuna, and in line with the notion that it represents an artificial grouping (Arnegard and Snoeks 2001), is rendered polyphyletic in all our analyses (figs. 2 and 3, supplementary fig. S1, Supplementary Material online). Therefore, the relation of *O. pachycheilus*, a rare deep-water species, to the other hypertrophied-lip taxa remains untested and could represent an additional origin of the hypertrophied-lip phenotype in Malawi cichlids. Likewise, if the recently described *T. pachychilus* (Dierickx et al. 2018) is indeed a congener of *Trematocranus placodon*, a species which was analyzed herein, this may represent yet another instance of convergent evolution of hypertrophied lips. Although logistically quite difficult to perform on this incredibly diverse radiation, a more quantitative assessment of the size and diversity of tissues contributing to the enlarged lips would further allow us to evaluate the degree of the repeated origins of these hypertrophied-lip phenotypes.

Several other more generalizable phylogenetic patterns emerged from our analyses of the genomes of the 86 species of closely related Lake Malawi cichlid fishes. SNPs obtained from whole-genome resequencing provided considerable power to test the monophyly of diagnosed taxa, particularly when concatenated for analysis with maximum likelihood in IQ-TREE. Yet, we obtained insufficient resolution for many of the relationships within the mbuna based on bootstrapping of the SVDquartet reconstructions. This stood in contrast to the high support across many of the mbuna estimated through ultrafast bootstrapping in IQ-TREE. Additionally, depending on the reconstruction method, the pelagic genera *Rhamphochromis* and *Diplotaxodon*, along with the possibly previously misidentified *Mylochromis lateristriga and Pallidochromis tokolosh*, comprise the earliest diverging group of the Malawi radiation (IQ-TREE) as has been found previously using mtDNA data (Meyer et al. 1994, 1996). However, these taxa are inferred to be either the sister to the nonmbuna or mbuna + *A. calliptera* clade based on SVDquartet analysis of noncoding or coding SNPs, respectively. The position of *Astatotilapia calliptera*, a haplochromine also found commonly outside of Lake Malawi, varied depending on reconstruction method and the SNP dataset used. In most analyses, *A. calliptera* is sister to the mbuna. Yet, in our SVDquartets tree derived from noncoding SNPs, *A. calliptera* was recovered as a sister to the rest of the Lake Malawi radiation (its traditional placement—Meyer et al. 1994, 1996; Malinsky et al. 2018). Although many genera were recovered as monophyletic with high support across analyses (i.e., *Buccochromis*, *Dimidiochromis*, *Labeotropheus*, *Nimbochromis*, and *Taeniolethrinops*), there are several taxonomic groupings that appear to be artificial taxonomic entities. *Placidochromis* is highly polyphyletic and several species exhibiting normal-sized lips (*Placidochromis electra* and *Placidochromis longimanus*) were placed outside of the “sand-dwelling” nonmbuna hypertrophied-lip clade, corroborating what has been reported in previous studies (Hulsey et al. 2018; Malinsky et al. 2018). *Lethrinops*, *Mylochromis*, and *Otopharynx* are also polyphyletic. Further, whereas IQ-TREE found a well-supported, distinct grouping of *Copadichromis* species with *Nyassachromis prostoma* nested among them, SVDquartets failed to recover any *Copadichromis* species together. A previous phylogenetic study by Hulsey et al. (2018) based on SNPs extracted from UCEs recovered a topology that does not strongly contradict that reported here for hypertrophied-lip species. This study, however, covered a narrower taxonomic sampling compared with that of our present investigation and in general provided much weaker topological support. The linkage among SNPs and whether they were protein-coding or noncoding was also not assessed for these UCEs.

### Comparisons Across Genomic Regions

**Fig. 4.**
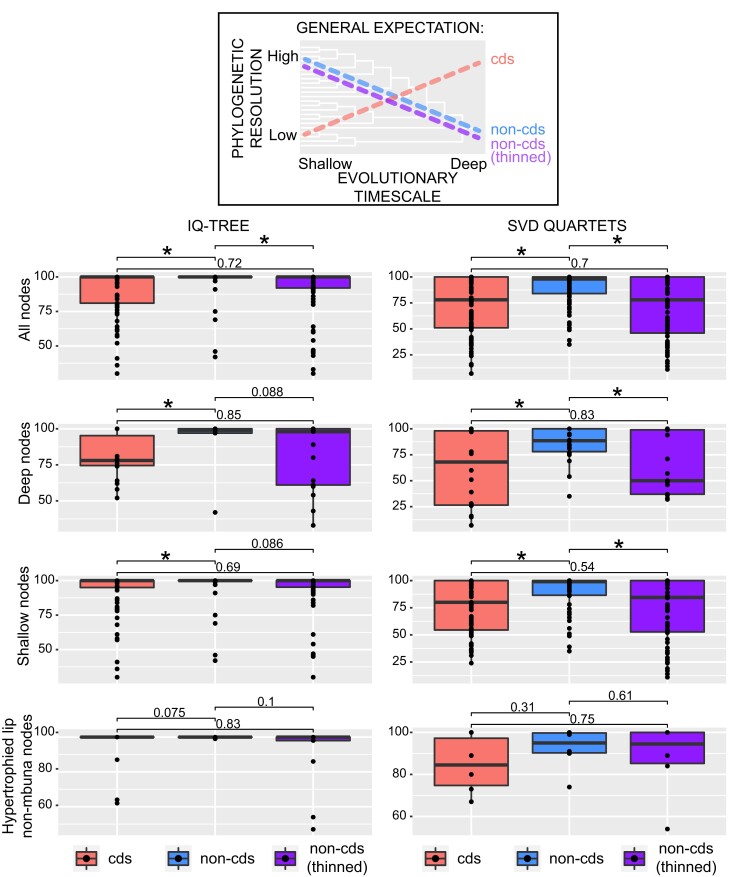
Expectations based on the literature of the resolving power of coding (cds) vs. unthinned and thinned noncoding (non-cds) SNPs across different nodal depths are depicted. The entire noncoding dataset contained ∼1.19 million SNPs. The thinned noncoding dataset was down-sampled to the same size as the coding dataset (∼50K SNPs). The different datasets are represented in all panels (cds = left boxplots, unthinned non-cds = middle boxplots, and thinned cds = right boxplots). In the left panels, the *y*-axis corresponds to ultrafast bootstrap values from the IQ-TREE analyses, and in the right panels, bootstrap support values from the SVDquartet analyses are shown with boxplots. Deep nodes refer to those in our phylogenies that have 10 or more nested terminal branches and shallow nodes are those that join fewer than ten tips. Ranges of support are also displayed exclusively for nodes within the clade of hypertrophied-lip nonmbuna (see node D, figs. 1 and 2). Significance was assessed using pairwise *t*-tests of mean support values (*α* = 0.05).

Given that coding regions are believed in general to carry less phylogenetic signal than faster-evolving noncoding regions among closely related lineages, we would expect coding SNPs to yield more poorly resolved trees (lower branch/node support values) for the Malawi radiation than those from noncoding regions. Additionally, we might expect noncoding SNPs to provide better resolution among more recently diverged lineages, especially between populations or species of Malawi cichlids that have diverged so recently (fig. 4). Thinning the number of noncoding SNPs to the same size data set as the coding DNA allowed us to evaluate whether these two data types provided fundamentally different phylogenetic signals or simply represent different sized data partitions of the genome. Despite these caveats, the distributions of node support derived from our IQ-TREE and SVDquartet analyses were largely similar when considering coding and noncoding datasets of equal size (∼50K SNPs) (fig. 4). Pairwise *t*-tests indicated no significant difference between the mean support values derived from these datasets. Furthermore, at both deep (≥10 nested terminals) and shallow (<10 nested terminals) nodes, the number and distribution of support values were comparable between these two data types. However, we did observe significant differences in resolution when comparing either of these smaller datasets to the entire noncoding SNP dataset (1,190,719 SNPs). In all, the much larger complete noncoding dataset provided better-resolved trees with fewer ambiguities. As coding SNPs appear to be as informative as noncoding site per site, incorporating SNPs from transcriptomic data for instance could contribute to the resolution of many rapidly radiating lineages including these Lake Malawi cichlids. However, the much greater amount of phylogenetically informative sites obtained from noncoding SNPs suggests that whole-genome resequencing might be a more powerful approach for fully resolving Malawi cichlid relationships as well as those of other large radiations. This approach is particularly useful for phylogenetic investigation in groups for which high-quality reference genomes exist and such resources are becoming more readily available, especially among vertebrates (Brawand et al. 2014; Conte et al. 2019; Kautt et al. 2020). Natural selection can influence the retention of standing genetic variation in both coding and noncoding sequences and hybridization can also drastically alter the genetic substrate exposed to selective forces (Seehausen 2004). Depending on which of these regions happen to be more greatly affected, discordance in tree topology between loci may arise and ultimately impact our ability to resolve clades. Given this caveat, it is likely advantageous to reconstruct phylogenies using both types of sequence data whenever possible.

### Gene Flow Among Hypertrophied-Lip Malawi Cichlids

Because hybridization might readily explain the appearance of hypertrophied lips in both mbuna and nonmbuna clades, we conducted tests for hybridization across the Lake Malawi fauna with Dsuite (Malinsky et al. 2021). This program applies ABBA–BABA tests to biallelic SNPs using sets of four taxa and assumes a pectinate tree topology usually denoted as ([{P1, P2}, P3], O). The outgroup (O) is used to define the ancestral allele (A) from the derived allele (B) and site patterns (i.e., BBAA, ABBA, and BABA) are counted across SNPs. Under the null model where only ILS is present (i.e., no gene flow, *D* = 0), ABBA–BABA patterns are expected to occur in equal frequency, but a significant divergence from this indicates potential introgression between P3 and either P1 or P2 (Malinsky et al. 2021). Across the entire dataset many (48,869×) significantly different (*P*-value < 0.001) ABBA–BABA patterns were found and the *D*-statistic values of these ranged from 0.2258 to 0.0071 (supplementary table S2 and fig. S3, Supplementary Material online). Similarly, Malinsky et al. (2018) also inferred numerous instances of gene flow within the Malawi radiation through the analysis of SNP data.

Yet, whereas introgression appears to be rampant across Lake Malawi species, *Abactochromis* and the hypertrophied-lip nonmbuna do not show elevated patterns of introgression with each other relative to other taxa. Nevertheless, of the 1,322 statistically significant trios involving potential hybridization with *A. labrosus*, 81 were suggestive of gene flow between *A. labrosus* and several hypertrophied-lip nonmbuna species (supplementary table S2, Supplementary Material online). However, of these, only three instances are represented among the highest 100 *D*-statistic scores calculated across all taxa. In general, the strongest evidence for gene flow was found to have occurred between the hypertrophied-lip taxa and other Lake Malawi species such as *A. calliptera* (supplementary fig. S3, Supplementary Material online).

### Comparisons With Other Lake Radiations and Future Directions

Most instances of convergent evolution have occurred in allopatric settings, but our results here add to the growing number of cases of replicate evolution that have arisen among closely related lineages inhabiting the same body of water (Muschick et al. 2012; Hulsey et al. 2019). Our results also highlight that Lake Malawi harbors multiple hypertrophied-lip species, whereas the other East African Great Lakes, Victoria, and Tanganyika, each has only one known species (Fryer and Iles 1972; Konings 2007; Oliver and Arnegard 2010). This disparity in species number may be due in part to Lake Malawi containing two distinct major radiations (the mbuna and nonmbuna) as well as the fact that the many isolated rocky reefs around the lake offer more extensive opportunities for adaptation and acquisition of novel phenotypes. Additionally, Lake Victoria, the youngest of the three lakes, has experienced the collapse of incipient species into hybrid swarms due to the loss of environmental heterogeneity and clear water habitats (Seehausen et al. 1997; Meier et al. 2017). This rampant hybridization could explain why hypertrophied-lip taxa have not evolved, or at least been inferred to have evolved, repeatedly, or speciated as seems to be the case for Lake Malawi. In contrast, given the relatively ancient age of Lake Tanganyika compared with Lakes Malawi and Victoria, additional hypertrophied-lip species may have gone extinct resulting in the single widespread species found today (*L. labiatus*). Because of the much longer timeframe over which the Tanganyikan radiation has evolved (Friedman et al. 2013; Irisarri et al. 2018), reduced gene flow among the more ancient lineages in Lake Tanganyika may have also played a role in limiting new hypertrophied-lip species from emerging across the phylogeny. Additionally, it is possible that the basic genetic architecture underlying the hypertrophied-lip phenotype is significantly different among cichlid lineages and even among these three lakes (Henning et al. 2017; Kautt et al. 2020). Adaptive introgression influencing the presence of enlarged lips, as a result, could be less likely depending on this trait’s (yet unknown) genetic complexity.

Future studies of the hypertrophied-lip evolution in Lake Malawi cichlids should focus more extensively on the genomic basis of this trait. These enlarged lips could be highly polygenic or they could result from changes in only one or two loci (Colombo et al. 2013; Manousaki et al. 2013; Henning et al. 2017; Kautt et al. 2020). The exact genomic architecture of the hypertrophied lips in Malawi might influence how likely a single hybridization event could have contributed to the repeated evolution of this trait across disparate phylogenetic lineages of Malawi cichlids (Riley et al. 2003; Mallet 2005; Taylor and Larson 2019). For instance, unlike traits that have been found to have a more polygenic basis such as body shape and pharyngeal jaw morphology, GWA mapping of lip size in Central American Midas cichlids has revealed high genomic associations at only two loci of major effect and the potential for introgression across multiple lineages (Kautt et al. 2020). A simpler genetic basis to lip size might generally allow it to readily introgress (Mallet 2005; Taylor and Larson 2019). Although introgression can play an integral role in shaping the genomic architecture of hybridizing lineages in adaptive radiations (Edelman et al. 2019), our current analyses provide no evidence that extensive interspecific introgression in Lake Malawi cichlids has contributed to the within-lake convergent evolution of hypertrophied lips.

## Conclusion

Within the Lake Malawi cichlid radiation, the evolution of hypertrophied lips has occurred multiple times. Even genome-wide protein-coding SNPs exhibited power to robustly reconstruct the relationships among Lake Malawi cichlids, but the much larger noncoding SNP dataset provided better-resolved inferences of relationships even within closely related Lake Malawi species. Both coding and noncoding phylogenomic reconstructions supported the monophyly of intraspecific sampling of several nonmbuna species with hypertrophied lips. These species also form a relatively closely related clade interspersed with a few other sand-dwelling nonhypertrophied-lip lineages. Additionally, our phylogenomic and comparative analyses coupled with tests for introgression are most consistent with hypertrophied lips having evolved independently in the sand-dwelling nonmbuna and rock-dwelling mbuna Lake Malawi cichlids. Future whole-genome-wide inference-based studies of Malawi cichlid relationships will continue to shed greater light on the patterns and processes of phenotypic and molecular evolution in this rapidly evolving adaptive radiation.

## Materials and Methods

### Whole-Genome Resequencing, Mapping, and Variant Discovery

We generated new whole-genome sequences for 33 individuals representing 24 cichlid species from Lake Malawi (supplementary table S1, Supplementary Material online). Our samples included five of the eight known Lake Malawi species with hypertrophied lips: *A. labrosus*, *C. euchilus*, *E. ornatus*, *P. milomo*, and *Placidochromis* “Mbenji fatlip”. High-molecular-weight DNA was extracted from fin or muscle tissue using a QIAGEN Dneasy Blood and Tissue Kit, whereas including an RNase A treatment step. DNA integrity was verified on agarose gels and concentrations were determined on a QuBit fluorometer. Genomic libraries were prepared with Illumina TruSeq DNA Nano kits targeting insert sizes of 350-bp and then paired-end sequenced (2 × 150 bp) on Illumina HiSeq platforms at the Beijing Genome Institute. Four individuals were pooled per lane with the aim of generating an approximate genome coverage of 20× per individual. Our genomic dataset was supplemented with additional short-read WGS data for 60 individuals sequenced by Malinsky et al. (2018) and seven individuals by Scherz et al. (2022) (supplementary table S1, Supplementary Material online).

Following demultiplexing, unmapped BAM files were generated from the raw FASTQs with Picard Tools v2.7.1 (*FastqToSam*), while marking Illumina adapters in the process (*MarkIlluminaAdapters*). Reads were then converted back to FASTQ format (*SamToFastq*) and mapped against the 22 chromosome assemblies of the latest version of the *Maylandia zebra* reference genome (GCA_000238955.5: M_zebra_UMD2a of Conte et al. 2019) using bwa -mem v0.7.17 (Li 2013). Metadata stored in the original unmapped BAM files were then added to the aligned BAM files using Picard *MergeBamAlignment* and PCR duplicates were annotated with Picard *MarkDuplicates*.

Variant discovery and genotype calling were preformed, whereas considering all samples together using freebayes v1.3.1 (Garrison and Marth 2012) and implementing standard quality filters (a minimum mapping quality 30 and a minimum base quality of 20). To speed up variant calling, we ran freebayes in parallel over separate 1 Mb regions spanning all 22 chromosomes and then concatenated the resulting VCFs into a single file with bcftools v1.3.1 *concat*. Hard quality filters were applied using the vcffilter script from the vcflib package (https://github.com/vcflib/vcflib) (command: -s -f “QUAL > 1 and QUAL/AO > 10 and SAF > 0 and SAR > 0 and RPR > 1 and RPL > 1”). The vt tools *normalize* and *uniq* (Tan et al. 2015) were then applied to normalize variants and remove duplicates. Further variant filtering was conducted with bcftools v1.11 to set individual genotypes with depth (“DP”) < 10× or >50× (approximately twice the raw mean depth per sample) and genotype quality (“GQ”) < 30 as missing (“./.”). We also included only SNPs with minor alleles present more than once (“MAC ≥ 2”), excluded any sites at which no alternate alleles remained after the filtering above (“AC == 0”) or where only alternate alleles were called (“AC == AN”), and removed possible false-positive singletons by excluding sites with a minor allele frequency of ≤5% (“MAF ≤ 0.05”). Lastly, we excluded sites with more than 20% missing data (“F_MISSING > 0.2”) and/or that were not biallelic SNPs (“-m2 -M2 -v snps”). This filtering scheme yielded a master VCF file containing 1,352,537 SNPs which was subsequently divided into separate datasets containing SNPs from noncoding and coding regions based on CDS annotations in the *M. zebra* reference genome (Conte et al. 2019). SNPs in these parsed datasets were further filtered based on linkage disequilibrium with bcftools 1.11-88 (Li et al. 2009; Danecek et al. 2021) using the +prune plugin. For each dataset, the squared correlation (*r*^2^) between alleles at each pair of loci within windows of 500 kb (+prune parameter –window 500,000) was calculated and highly linked SNPs (*r*^2^ > 0.9; +prune parameter –max 0.9) were discarded. This reduced the size of the noncoding and coding datasets to 1,190,719 and 54,021 SNPs, respectively, which were lastly converted into NEXUS and PHYLIP formatted alignments with IUPAC ambiguity codes applied to heterozygous sites using the python script vcf2phylip.py (Ortiz 2019) (https://github.com/edgardomortiz/vcf2phylip).

### Phylogenomic Analysis

To assess which hypertrophied-lip species of cichlids in Lake Malawi compose monophyletic groups, phylogenetic analyses were conducted on the noncoding and coding datasets with *Astatotilapia bloyeti* (a non-Lake Malawi species) designated as an outgroup. Maximum likelihood trees were inferred from the SNP datasets using IQ-TREE v1.6.12 (Nguyen et al. 2015). For this analysis, the ascertainment bias correction was applied to correct for the absence of invariant sites in the sequence alignment (command -m MFP + ASC) (Kalyaanamoorthy et al. 2017) and 1,000 ultrafast bootstrap replicates (command -bb 1000; UFBoot Minh et al. 2013) were preformed to assess branch support.

To augment our inferences of relationships, we also used SVDquartets (Chifman and Kubatko 2014) as implemented in PAUP* v4.0a166 (Swofford 2002) to reconstruct the species tree from the noncoding and coding datasets under its coalescent-based framework. Unlike summary methods that rely on a priori reconstructed gene trees to estimate the species tree, this program uses sequence data directly to infer quartet trees and performs well even in the presence of gene flow (Long and Kubatko 2018). For our analysis, the multispecies coalescent tree model was selected and individual samples were each assigned to a taxon partition (i.e., their respective species). We exhaustively sampled all quartets (3,751,519 in total) and inferred the species tree using the Quartet FM algorithm (QFM; Reaz et al. 2014). Subsequently, one hundred bootstrap replicates for each data partition were carried out to assess branch support.

After obtaining reconstructions from these analyses, we compared the resolving power of coding vs. noncoding SNPs at various nodal depths. Ultrafast bootstrap values from IQ-TREE and bootstrap support values from SVDquartet were compared across the analyses conducted with the coding and noncoding datasets. For a fair comparison between the contrasting datasets, we thinned the VCF file containing only the noncoding SNPs to a size comparable to that of the coding dataset (∼×50K SNPs) using the bcftools + prune plugin. For these comparisons, nodes that have ten or more nested terminal branches were referred to as “deep” and those with fewer than ten tips as “shallow.” Significance was assessed using pairwise *t*-tests of mean support values (*α* = 0.05) as implemented by the “stat_compare_means” function of the R package *ggpubr* (Kassambara 2020).

Maximum likelihood reconstruction of ancestral states was performed using the “fastAnc” function of RStudio package *phytools* (Revell 2012). The presence and absence of hypertrophied lips were categorized as a discreet variable and the transition probabilities between these two states were considered to be equal. The probabilities of lineages possessing either hypertrophied lips or not at each node for the noncoding SNP-based tree derived with IQ-TREE are displayed in supplementary fig. S2, Supplementary Material online and are represented for relevant nodes as pie-diagrams in figure 2.

### Analysis of Gene Flow

Because we found hypertrophied-lip cichlid species of Lake Malawi to fall within both the mbuna and nonmbuna radiations, we investigated their genomic histories further. To assess the degree of interspecific gene flow across all ingroup taxa including that between hypertrophied-lip species, we calculated genome-wide Patterson’s *D* (ABBA/BABA) statistics as implemented in the program Dsuite (Malinsky et al. 2021). This test is applied to biallelic SNPs across four taxa and assumes a pectinate tree topology ordered as ([{P1, P2}, P3], O). The outgroup (O) helps to define the ancestral allele (A) from the derived allele (B) and site patterns (BBAA, ABBA, and BABA) for each SNP are counted. Under the null model where only ILS is present (i.e., no gene flow, *D* = 0), ABBA–BABA patterns are expected to occur with equal frequency, but a significant divergence from this indicates that introgression may have happened between P3 and either P1 or P2 (Malinsky et al. 2021). Using the 1,352,537 SNPs from the master VCF file and *A. bloyeti* set as the outgroup, we assessed all possible three taxon combinations (102,340 in total) with the “Dtrios” function. Each trio was ordered so that the BBAA pattern was maximized in the output. Standard jackknife blocks (×20) were used to determine if the resulting *D*-statistic values differed significantly from zero. To account for multiple tests, *P*-values were adjusted in RStudio 4.0.3 by applying the false discovery rate method of Benjamini and Hochberg (1995) with the *stats* package (command: p.adjust [*P*_values, method = “fdr”]). An *α* of 0.001 was applied to identify statistically significant *D*-statistic values. To visualize species pairwise comparisons of *D*-statistic scores, a heatmap was generated using the Ruby script plot_d.rb (available at: https://github.com/mmatschiner).

## Supplementary Material

Supplementary data are available at *Genome Biology and Evolution* online.

## Supplementary Material

evac051_Supplementary_Data

## Data Availability

Raw whole-genome resequencing reads for the 33 newly sequenced taxa have been deposited in NCBI’s Sequence Read Archive (see supplementary table S1, Supplementary Material online). Tree files obtained from our analyses are available as Supplementary material, Supplementary Material online.
