# Supplementary material for: Phylogenomic Analyses Show Repeated Evolution of Hypertrophied Lips Among Lake Malawi Cichlid Fishes
Source: Genome Biol Evol. 2022 Apr 13;14(4):evac051. doi: 10.1093/gbe/evac051 (PMC9017819; doi:10.1093/gbe/evac051)
Supplement: evac051_Supplementary_Data [file evac051_supplementary_data.zip › Masonicketal_SupplementaryMaterial.docx]

**Phylogenomic analyses show repeated evolution of hypertrophied lips among Lake Malawi cichlid fishes**

Paul Masonick^1^, Axel Meyer^1^, C. Darrin Hulsey^1,2^

^1^ Department of Biology, University of Konstanz, Universitätsstraße 10, 78464 Konstanz, Germany

^2^Current Address: School of Biology and Environmental Science, University College Dublin, Belfield, Dublin 4, Ireland.

Corresponding author: Darrin Hulsey

E-mail: [darrin.hulsey1@ucd.ie](mailto:darrin.hulsey1@ucd.ie)

**Supplementary Materials**

This file includes:

Supplementary Figures 1–3

*Supplementary Tables 1–2 are provided as separate Excel files.

**Supplementary Figures**


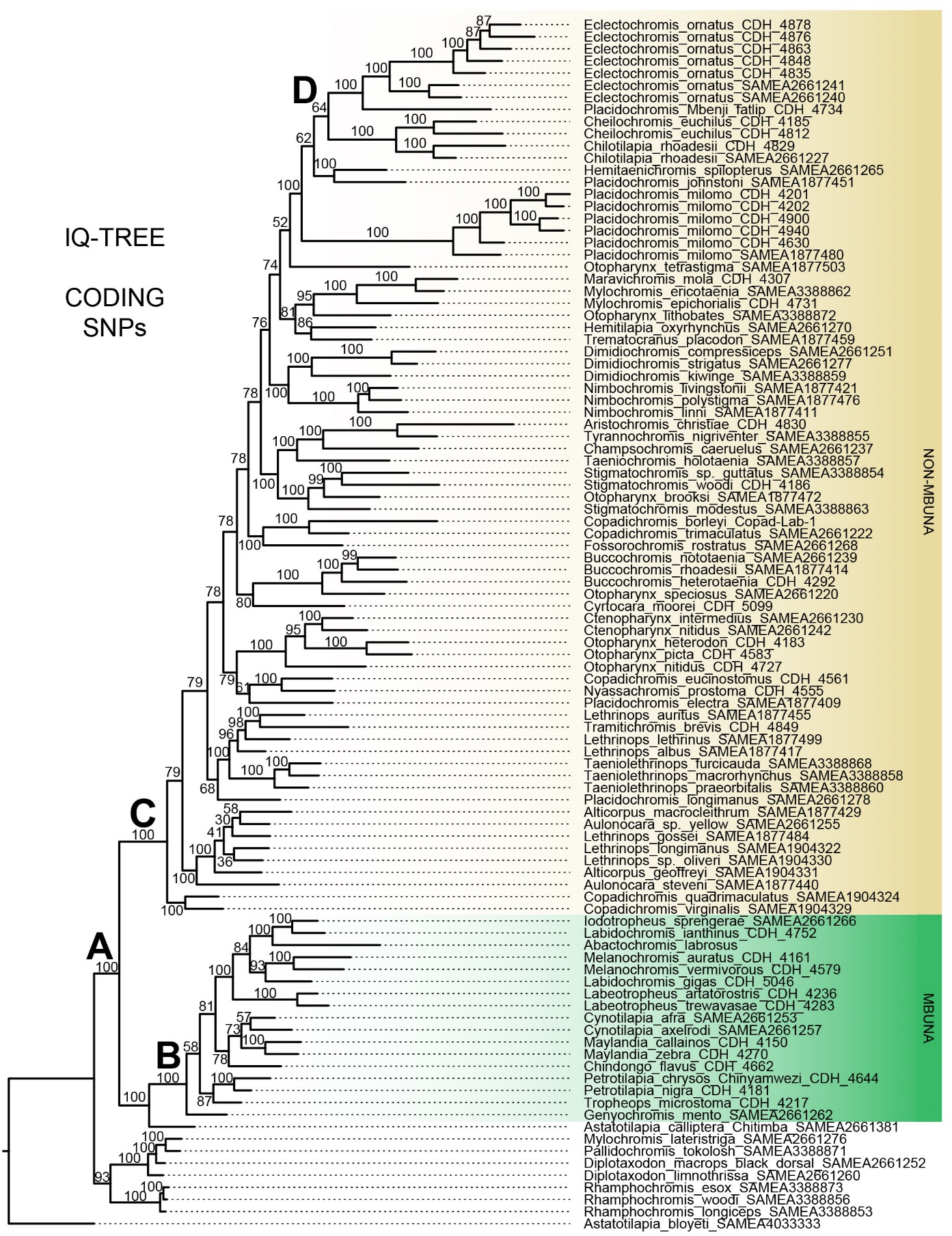


Supplementary Figure 1.

Maximum likelihood IQ-TREE species tree reconstruction of Lake Malawi cichlids based on the coding SNP dataset. Ultrafast bootstrap support values are given for each branch. Node A: MRCA of all Lake Malawi hypertrophied species, node B: MRCA of the mbuna radiation, node C: MRCA of the haplochromine non-mbuna, node D: MCRA of the hypertrophied lip non-mbuna.


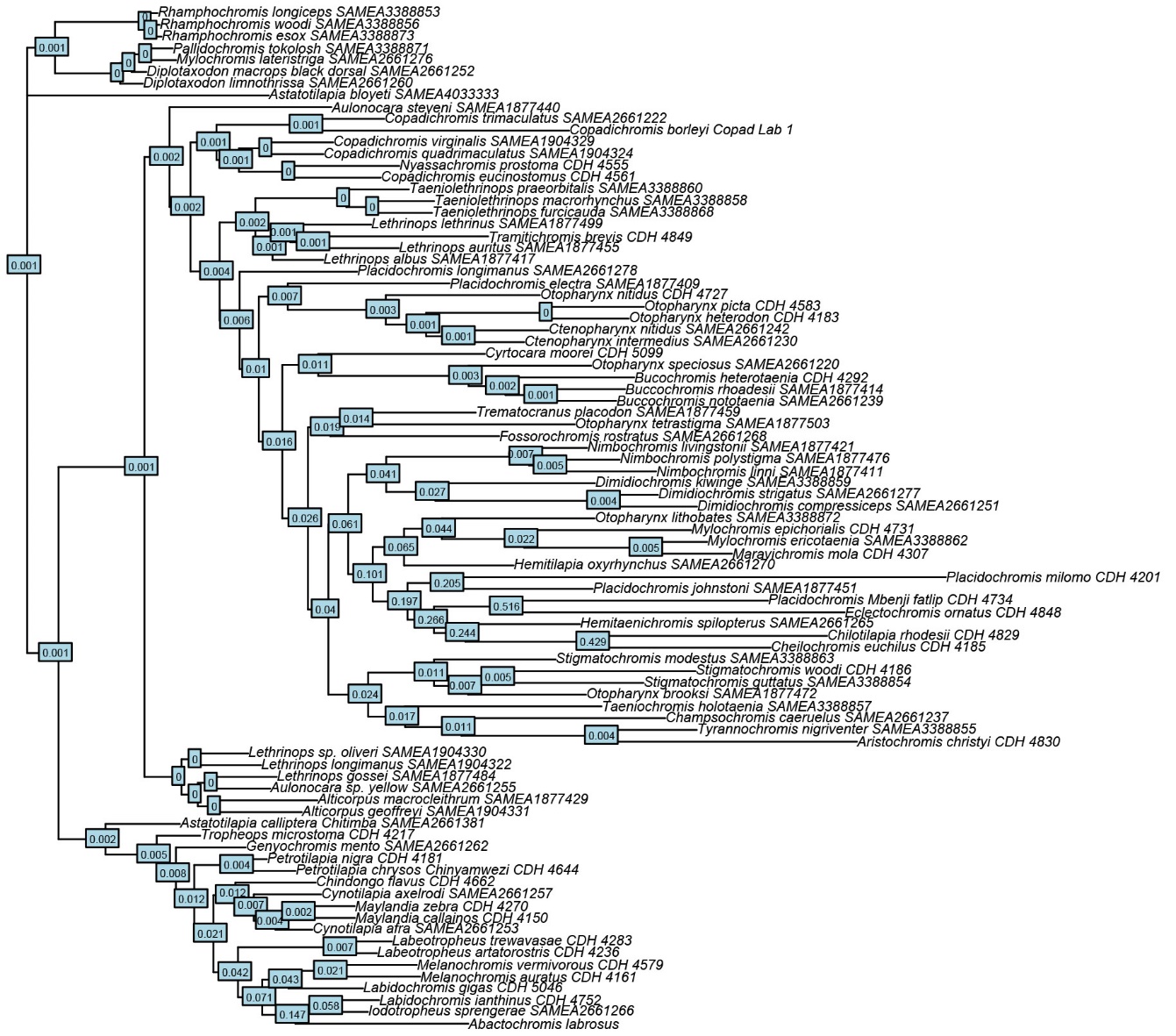


Supplementary Figure 2.

Ancestral state reconstruction of hypertrophied lips. Maximum likelihood reconstructions were conducted across the non-coding SNP-based IQ-TREE phylogeny (of Figure 1) using the ‘fastAnc’ function of the R package *phytools* (Revell 2012). The presence and absence of hypertrophied lips was categorized as a discreet variable and the transition probabilities between these two states were considered to be equal. The probability that the ancestor for each tested node had hypertrophied lips is indicated.


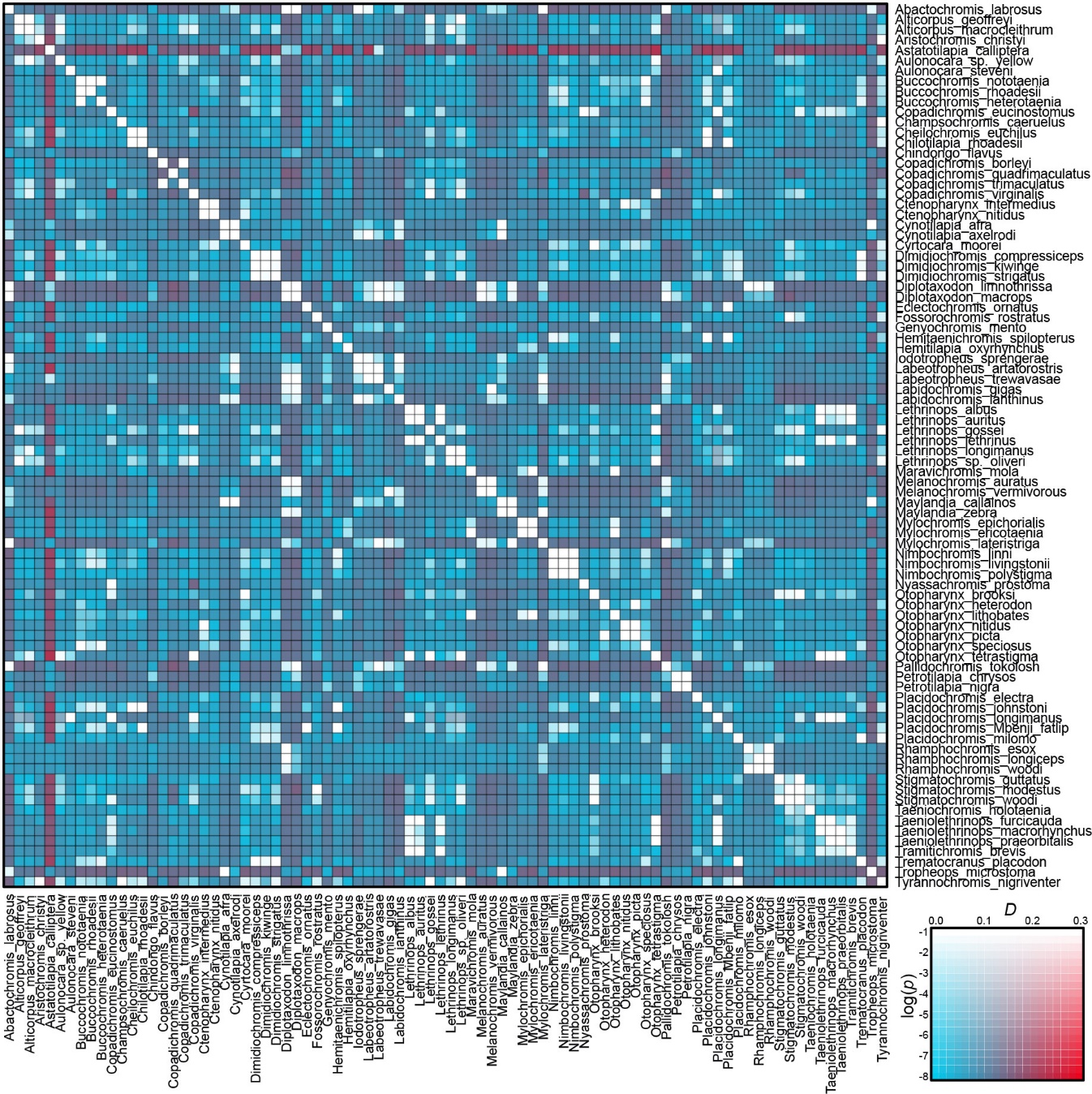


Supplementary Figure 3.

Introgression analysis heatmaps depicting pairwise comparisons across taxa for Patterson’s *D*-statistic (ABBA-BABA) values calculated in the program Dsuite (see Supplementary Table 2 for statistical information). The most significant values found between two species is illustrated as a color with warmer colors suggesting an increased possibility that gene flow has occurred. While cases of gene flow are inferred between some taxa, these tests failed to detect elevated signals of introgression between *Abactochromis labrosus* and the hypertrophied lip non-mbuna.

**Supplementary Tables**

Supplementary Table 1. Whole genome resequencing specimen voucher information and accession numbers.

Supplementary Table 2. (A) Significant *D*-statistic scores involving introgression between *Abactochromis* (in bold) and other Lake Malawi taxa (*p*<0.001). Yellow highlights denote introgression between hypertrophied lip species. (B) Significant *D*-statistic scores calculated from ABBA-BABA tests across all Lake Malawi trios (*p*<0.001).
